# MicroRNAs in opioid addiction: elucidating evolution

**DOI:** 10.3389/fgene.2012.00241

**Published:** 2012-11-26

**Authors:** Emily J. Wood, Leonard Lipovich

**Affiliations:** Center for Molecular Medicine and Genetics, School of Medicine, Wayne State UniversityDetroit, MI, USA

**Keywords:** microRNA, morphine, evolution, TargetScan, OPRM1

## Abstract

Three reviews in the Frontiers Research Topic “Non-Coding RNA and Addiction” (He and Wang, 2012; Rodriguez, 2012; Zheng et al., 2012), grouped under the chapter “MicroRNAs and Morphine,” focus on the contribution of microRNAs to opioid abuse. Although animal models have been fundamental to our understanding of addiction pathways, the assumption that microRNAs implicated in opioid tolerance – and their binding sites in mRNAs – are conserved in mammalian evolution was not examined by the authors. Inspired by recent reports which highlight a surprising lack of evolutionary conservation in non-coding RNA genes, in this perspective we use public genome, annotation, and transcriptome datasets to verify microRNA host gene, mature microRNA, and microRNA binding site conservation at key loci functional in opioid addiction. We reveal a complex evolutionary landscape in which certain directional regulatory edges of the microRNA–mRNA hub-and-spoke network lack pan-mammalian conservation.

## INTRODUCTION

Although microRNAs are not the most abundant class of non-coding RNAs in mammalian systems, numbering merely 2,042 mature sequences^1^ mirbase.org compared to the more than 9,000 long non-coding RNA genes in humans (Derrien et al., 2012), microRNAs represent a key category of post-transcriptional suppressors, and their global significance in gene expression regulation is firmly established (Bartel, 2009). Nevertheless, evolutionary conservation of protein-coding genes exceeds that of non-coding RNA genes (Derrien et al., 2012). Mammalian microRNA host genes are structurally heterogeneous and highly complex, with variable localization of the mature microRNA sequences relative to key host gene structure elements such as promoters, 3′ends, exons, splice sites, and introns (Rodriguez et al., 2004). Evolutionary complexity may accompany this genomic structure complexity of microRNA host genes: a microRNA cluster functional in stem cell regulation and pluripotency resides within a non-coding RNA host gene that exists only within placental mammals and exhibits major genomic structure distinctions between closely related species (Houbaviy et al., 2005). By no means confined to early development, sequence non-conservation of microRNA-dependent regulation is increasingly apparent in the brain. Brain-expressed microRNAs and their mRNA targets functional in neural development display accelerated rates of evolutionary change along the human lineage (Somel et al., 2011), while expression differences, some of which are shaped by adaptive forces, are evident among the microRNA repertoires of human, chimpanzee, and macaque (Dannemann et al., 2012). In summary, this evidence for gene structure as well as sequence non-conservation of microRNA host genes, mature microRNAs, and microRNA:mRNA hybridization sites motivated us to systematically examine the conservation of microRNA and target sequences implicated in opioid abuse and tolerance.

## METHODS

We used the UCSC Genome Browser^2^(hg19 human genome assembly), miRbase^3^, and the NCBI website^4^, accessed on May 25 and September 20, 2012.

## RESULTS AND DISCUSSION

Some microRNAs prominent in the drug abuse field, as well as their host genes and mRNA targets, lack gene structure conservation and sequence conservation within mammals. Because functional genomics of drug abuse continues to depend on animal, especially rodent, models, and because of the accumulating evidence in the literature regarding non-conservation and rapid evolution of microRNA genes, it is imperative to rigorously test the assumption that the key microRNA-to-mRNA regulatory relationships implicated in human drug abuse can, in fact, be extrapolated to animal models. We tested this assumption for microRNAs from **Table 1** at three levels: (1) genomic conservation of the mature microRNA’s seed (nt 2–nt 8) sequence within the microRNA host gene; (2) gene structure conservation of the microRNA host gene; and (3) genomic conservation of the microRNA’s binding site in the 3′UTR of the known mRNA target from the three reviews (**Table 2**). In examining microRNA seeds and host genes, we focused only on three microRNAs (not including let-7) whose names were sufficiently specific to allow *in silico* identification of their host gene loci in the UCSC Genome Browser: miR-23b, miR-133b, and miR-190. We found that two of the three microRNAs resided within protein-coding host genes, while one host gene was non-coding and had only expressed sequence tag (EST) evidence for a conventional, capped, polyadenylated host gene transcript. We also identified substitutions in the seed sequence of all three microRNAs in mammalian species from the public genome-wide 46-species MultiZ alignment, suggesting that the affinity of all three microRNAs for their known targets may differ between mammalian species, unless the target UTRs co-evolve with the microRNA seeds. Gene structure conservation of the miR-190 host gene, as assessed by the conservation of the canonical 3′end polyadenylation signal AnTAAA hexamer in the 46-species alignment was also incomplete, with two of the three host genes in human apparently reliant on primate-specific polyadenylation signals (**Table 2**).

**Table 1:** MicroRNAs and morphine: function and conservation – MicroRNA names, functions, and targets from the “MicroRNAs and Morphine” chapter of the Frontiers Special Topic “Non-Coding RNA and Addiction.”

Name	Review	Description from review and target
miR-23b	Zheng et al. (2012)	Targets the 3′-UTR of OPRM1 mRNA and regulated the association between OPRM1 mRNA and polysomes
miR-23b	He and Wang (2012)	Could interact with the MOR 3′-UTR via a K box motif (5′-UGUGAU-3′) in SH-SY5Y and mouse P19 cells
miR-23b	Rodriguez (2012)	Involved in linking MOR expression and morphine treatment at the post-transcriptional level. Represses MOR through 3′ UTR of MOR1 in K-box motif
miR-133b	Zheng et al. (2012)	Morphine reduces miR-133b and miR-133b increases Pitx3
miR-133b	He and Wang (2012)	Decreased by morphine in zebrafish embryos
miR-133b	Rodriguez (2012)	Possible involvement in addiction through the effects of morphine, Targets Pitx-3 (Th and Dat pathway), miR-133b also down-regulates RhoA protein expression
miR-190	Zheng et al. (2012)	Negatively regulated by fentanyl; decreased expression of miR-190 leads to an increase in NeuroD protein. miR-190 binds to the 3′UTR of NeuroD mRNA and destroys it
miR-190	Rodriguez (2012)	Targets NeuroD levels, and is negatively regulated by fentanyl. miR-190 (aka miR-190a?) regulates NeuroD, a transcription factor that is known to regulate the differentiation and maturation of neurons
miR-let7	Zheng et al. (2012)	Morphine treatment affected the expression levels of miR-23b and let-7, which have binding sites on the 3′UTR of the OPRM1 mRNA and control the expression of OPRM1
miR-let7	He and Wang (2012)	Target is 3′ end of MOR mRNA. These authors experimentally validated the *in silico* prediction that members of the let-7 miR family can interact with the 3′-UTR of MOR mRNA at the predicted positions
miR-let7	Rodriguez (2012)	Works as a mediator of the movement of the mu opioid receptor (MOR) mRNA into P-bodies, which leads to translational repression of MOR mRNA. Targets MOR through 3′ UTR binding

**Table 2:** MicroRNAs and morphine: function and conservation – Seed sequence, host gene, and target binding site conservation of microRNAs with known opioid abuse functions, as assessed by viewing the corresponding microRNA host genes and their mRNA targets within the genome-wide 46-species MultiZ alignment in the human UCSC Genome Browser.

MicroRNA name	MicroRNA host gene name	mRNA target of the microRNA	Conservation of microRNA host gene	Closest species to human lacking 100% miR seed conservation at the mRNA target’s binding site
miR-23b	C9ORF3	IPCEF1 and OPRM1	Vertebrates, but polyA signal is primate-specific	Gorilla
miR-190	TLN2	NEUROD1	Vertebrates	Mouse
miR-133b	Novel host (ESTs: AI803441 and others)	PITX3	Vertebrates, but polyA signal is primate-specific	n/a (not detected by TargetScan)

**FIGURE 1:**
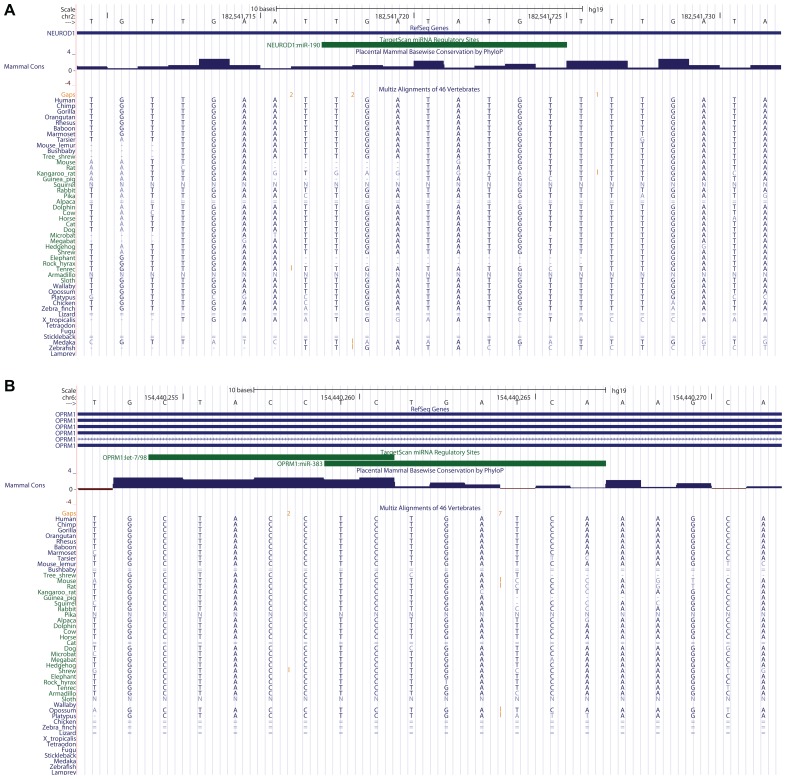
**MicroRNA binding sites at opioid addiction genes’ 3′UTRs: conservation and complexity**. **(A)** The miR-190 binding site in the human NeuroD1 3′UTR is not conserved in the mouse, according to the 46-species MultiZ sequence alignment in the UCSC Genome Browser. **(B)** Let-7 and miR-383 binding sites overlap in the OPRM1 3′UTR.

We proceeded to gage the conservation of these microRNAs’ binding sites in the 3′UTRs of their known mRNA targets, by visualization of the TargetScan microRNA target prediction tool results that are represented in the “TS miRNA” track of the human UCSC Genome Browser. We focused on three target relationships, which are reported in the three review articles comprising our “MicroRNAs and Morphine” chapter of the present Research Topic: miR-23b/OPRM1, miR-190/NeuroD, and miR-133b/Pitx3. We observed the potential absence (due to a genomic deletion relative to human and/or to a genome assembly gap in the non-human species) of two microRNA binding sites at these mRNA targets in at least one non-human mammal. The miR-190 binding site of the human NeuroD1 3′UTR is not conserved in mouse, a key animal model for addiction research used to study the OPRM1-NeuroD crosstalk via miR-190 (**Figure 1A**; Rodriguez, 2012 and references therein). We could not evaluate the third site, the miR-133b-binding sequence of the Pitx 3′UTR, because this well-known relationship is not predicted by the pre-computed TargetScan results that are made available in the TS/miR track of the UCSC Genome Browser (hg19 assembly). This deficiency attests to a false-negative rate in microRNA target prediction tools that results in the omission of some experimentally confirmed microRNA targets by these tools. Similarly, TargetScan did not detect the reported miR-23b targeting of the OPRM1 3′UTR, although, intriguingly, the neighboring gene IPCEF1 – which overlaps, in an antisense orientation, the genomic footprint of one OPRM1 splice isoform – is a miR-23b target (Table 2). These results summarily indicate that microRNA host gene structures, mature microRNA seed sequences, and microRNA binding sites at mRNA 3′UTRs are not always conserved in all mammals, for some of the key microRNAs and their experimentally confirmed mRNA targets in opioid abuse.

Manual annotation of microRNA:mRNA cognate sites may uncover novel regulatory relationships relevant to drug abuse transcriptomics. In the process of assessing sequence conservation at TargetScan-predicted, experimentally confirmed microRNA binding sites at loci of importance to opioid addiction, we often observed that numerous additional microRNAs – not mentioned in the literature concerning post-transcriptional regulation at these loci – are predicted by TargetScan to regulate these same targets. In fact, 16 distinct microRNA binding sites are predicted by TargetScan in the NeuroD1 3′UTR alone. In several particularly interesting cases, two microRNA binding sites partially overlap within a single target gene’s 3′UTR. This overlap implies that a given mRNA of that gene may be suppressed by either of the two microRNAs competing for the binding site, but may not be targeted by both microRNAs simultaneously. One intriguing example of these overlaps is found in the 3′UTR of OPRM1, where the experimentally validated let-7 microRNA binding site overlaps a TargetScan-predicted site for miR-383 (**Figure 1B**). This indicates the utility of UCSC Browser manual annotation in discovering possible new microRNA binding sites, which should be experimentally evaluated for function. These overlapping sites are also intriguing within the context of the competing endogenous RNA (ceRNA) hypothesis (Tay et al., 2011), since one of them in theory competes with the other for binding the same target.

## CONCLUSION

We examined the evolutionary conservation of microRNA host genes and experimentally confirmed microRNA targets, focusing on validated microRNA:mRNA regulatory relationships that are central to gene expression regulation in the context of opioid addiction. We pinpointed a lack of pan-mammalian conservation at multiple levels. In particular, microRNA host genes contained primate-specific genomic structure elements, such as consensus polyadenylation signals that are absent beyond primates. At the same time, TargetScan-detected, experimentally validated microRNA seed binding sites at mRNA 3′UTRs lacked pan-mammalian conservation; certain seed matches in 3′UTRs were not even conserved in mouse. This evolutionary complexity reveals the potential limitations of animal models in which these protein-coding genes might be regulated by microRNAs differently from human. High-throughput short and long RNA sequencing should facilitate future studies of microRNA roles in drug addiction, through quantitating events such as alternate polyadenylation that are essential for making microRNA binding sites available in 3′UTRs, and through unbiased analysis of co-expression and co-localization of annotation-identified new microRNAs (e.g., miR-383) with their putative mRNA targets.

## Conflict of Interest Statement

The authors declare that the research was conducted in the absence of any commercial or financial relationships that could be construed as a potential conflict of interest.

## Footnotes

1 mirbase.org

2 genome.ucsc.edu

3 mirbase.org

4 www.ncbi.nlm.nih.gov
